# Older adults use a motor plan that is detrimental to endpoint control

**DOI:** 10.1038/s41598-021-86959-9

**Published:** 2021-04-07

**Authors:** Stefan Delmas, Yoon Jin Choi, Marcel Komer, Michelle Weintraub, Basma Yacoubi, Evangelos A. Christou

**Affiliations:** 1 Department of Applied Physiology and Kinesiology, University of Florida, Gainesville, FL 32611 USA; 2 Department of Neurology, Norman Fixel Institute of Neurological Disorders, University of Florida, Gainesville, FL 32611 USA

**Keywords:** Ageing, Neurophysiology

## Abstract

Here, we aimed to understand if older adults (OA) use a unique motor plan that is detrimental to endpoint control. We performed two experiments that used ankle ballistic contractions that reversed at the target. In Experiment 1, eight young adults (YA; 27.1 ± 4.2) and eight OA (73.3 ± 4.5) aimed to perform an ankle dorsiflexion–plantarflexion movement that reversed at 9° in 180 ms (target). We found that the coordination pattern (motor plan) differed for the two groups. OA used significantly greater soleus (SOL) activity to reverse the ankle movement than YA and exhibited greater tibialis anterior (TA) muscle activity variability (p < 0.05). OA exhibited worse endpoint control than YA, which associated with the exacerbated TA variability (R^2^ > 0.2; p < 0.01). Experiment 2 aimed to confirm that the OA motor plan was detrimental to endpoint control. Fifteen YA (20.5 ± 1.4) performed an ankle dorsiflexion–plantarflexion contraction that reversed at 30% MVC in 160 ms by using either a pattern that mimicked OA (High SOL) or YA (Low SOL). With the High SOL coordination pattern, YA exhibited impaired endpoint control and greater TA activation variability. These findings provide strong evidence that OA select a unique motor plan that is detrimental to endpoint control.

## Introduction

The control of movement endpoint is necessary for the successful execution of many activities of daily living. For example, driving necessitates fast but accurate movement of the foot from the gas pedal to the brake pedal. This type of movement, which is used extensively during car following in traffic, requires extraordinary endpoint accuracy of the foot in terms of space, timing, and force. There is strong evidence that aging increases movement errors and variability, which impair endpoint control^1–4^. The current hypothesis is that such age-related impairments relate to changes in the timing parameters of the motor plan^1,2,4,5^. An important but unresolved question is whether age-associated differences in motor planning extend beyond timing characteristics and are affected by the selection of a unique motor plan to accomplish targeted movements. Here, to address this question, we performed two experiments. In the first experiment, we compared the motor plan and movement endpoint control of ballistic targeted contractions between young and older adults. In the second experiment, we compared movement endpoint control when young adults performed ballistic targeted contractions with a motor plan that mimicked older adults and a motor plan that mimicked young adults.

We selected an experimental model that incorporates ballistic submaximal targeted contractions, defined as contractions that are not influenced by online sensorimotor corrections, for the following two reasons: First, age-associated differences in motor control are more robust for ballistic targeted contractions compared with tracing contractions. Older adults exhibit greater endpoint error and variability anywhere from 10 to 90% MVC^6^. In contrast, age-associated differences in tracing tasks (slower movements) typically occurs for contractions at lower force levels (< 10% MVC) and the results are heavily influenced by the magnitude of visual feedback^6^. Second, the endpoint of ballistic contractions is a good experimental model to study age-associated differences in the motor plan. Such contractions are not influenced by visual or proprioceptive online feedback, as there is not sufficient time to integrate and adjust the executed motor command (online corrections)^5,7–10^. Rather, the endpoint of ballistic contractions is influenced by the pre-selected motor plan and the motor plan adjustments that occur from trial to trial. Therefore, age-associated differences in endpoint control during targeted ballistic contractions largely depend on the motor plan.

Recently, we have demonstrated that muscle coordination during ballistic targeted contractions differs for young and older adults, independent of movement kinematics and individual activity of the involved muscles^5^. Specifically, we found that when young and older adults perform ballistic ankle movements that must reverse at the target (dorsiflexion–plantarflexion), older adults exhibit an increased time delay between the activation of the dorsiflexor (tibialis anterior) and plantarflexor (soleus) muscles. We have shown that a longer time delay between the dorsiflexor and plantarflexor muscles relates to the age-associated differences in endpoint control^1,2,4^. However, it remains unknown if the motor plan differences between young and older adults are broader and include other parameters, such as the relative contribution in amplitude of muscle activity.

To test the hypothesis that older adults select a unique motor plan that is detrimental to movement endpoint control we performed two experiments. As in previous studies^1–3,5,11^, we used movements that involved a single joint (ankle) and self-initiated discrete ballistic targeted contractions that reversed at the target. In the first experiment, we aimed to determine differences in the motor plan and its effect on movement endpoint control of young and older adults. In the second experiment, we recruited a distinct group of young adults and compared their movement endpoint control while performing ballistic targeted contractions using two unique motor plans. The motor plans mimicked the dorsiflexion–plantarflexion muscle coordination used by young or older adults from experiment 1.

## Methods

### Experiment 1

#### Participants

Eight young adults (mean 27.1 ± 4.2 years, range 23–35 years, 3 women) and eight old adults (mean 73.3 ± 4.5, range 69–82, 5 women) volunteered to participate in this study. All participants reported being healthy without any known neurological or orthopedic disorders. Participants were right-handed^12^ and right-footed^13^. Before participating in the study, all participants signed a written, informed consent approved by the Institutional Review Board at the University of Florida. Accordingly, all procedures were approved by the Institutional Review Board and performed in accordance with the Declaration of Helsinki.

#### Experimental approach

Participants completed one testing session that lasted ~ 1 h in which they performed goal-directed ankle movements with their non-dominant limb. Testing on the non-dominant limb provides a model that is less affected by previous experience^14^ and is consistent with prior studies of neuromuscular control^1^. At the beginning of each session, we explained the experimental procedures and the goal-directed task (ankle joint movement) to the subjects. Each subject performed the following procedures within a session: (1) maximal voluntary contraction (MVC) tasks with ankle dorsiflexion/plantarflexion; (2) practice of 3–5 goal-directed movement trials at a target different from the actual target; (3) 100 goal-directed movement trials with the ankle, and (4) repetition of the MVC task.

#### Experimental setup

Each participant was seated comfortably in an upright position and faced a 32 inch monitor (Sync Master 275t+, Samsung Electronics America) that was located 1.25 m away at eye level. The monitor displayed the ankle movement produced with a custom-written program in MATLAB (MathWorks, Natick, MA). All subjects affirmed that they could see the display clearly. The left hip joint was flexed ∼ 90° with 10° abduction, the knee was flexed to ∼ 45°, and the ankle was plantarflexed to ∼ 15°. The normal range of dorsiflexion from a flat position is 0–15°^15^. However, ankle joint flexibility decreases by 35–50% in older adults^16^. Thus, we opted for a 15° plantarflexed starting position to increase the available degrees of dorsiflexion so participants do not feel overly strained while performing the task. The left foot rested on a customized foot device with an adjustable footplate that we secured by straps over the metatarsals to ensure a secure position and simultaneous movement between the device and the foot. We positioned the axis of rotation of the customized foot device in line with the axis of rotation of the left ankle. This arrangement allowed only dorsiflexion and plantarflexion of the ankle. In this study, we focused on the tibialis anterior and soleus muscles, which contribute significantly to dorsiflexion and plantarflexion.

#### Measurements

##### Ankle displacement

The displacement of the ankle (dorsiflexion) was measured with a low-friction potentiometer (SP22G-5K, Mouser Electronics, Mansfield, TX) that was located directly lateral to the fibular malleolus. The ankle position signal was sampled at 1000 Hz with a NI-DAQ card (model USB6210, National Instruments, Austin, TX) and stored on a personal computer.

##### EMG

Muscle activation was recorded with a Trigno wireless EMG system (Delsys, Boston, MA) from the tibialis anterior and soleus muscles during the ankle dorsiflexion task. We placed the recording electrodes on each muscle on the skin and in line with the muscle fibers. The location for each electrode was selected according to the European Recommendations for Surface Electromyography^17^. All EMG signals were sampled at 1 kHz with NI-DAQ board (Model USB6218, National Instruments, Austin, TX, USA) and stored on a personal computer.

#### Experimental tasks

##### Maximal voluntary contraction (MVC) task

We measured the MVC for ankle dorsiflexion and ankle plantarflexion as we have done so previously^18–20^. Subjects increased force to their maximum in 3 s and maintained the maximal force for ∼ 3 s. They exerted three to five MVCs or until two MVC trials were within 5% of each other. We provided one-minute rest between consecutive trials. In addition, we recorded the peak EMG (average of 0.5 s around the peak EMG of the trial) during MVC, which was used to normalize the EMG during the goal-directed movements.

##### Goal-directed task

**Figure 1:**
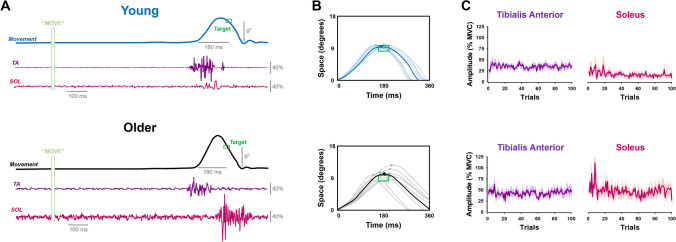
Movement endpoint control and muscle activation in young and older adults. Participants performed a goal-directed ankle dorsiflexion movement with the non-dominant foot that was restrained in an ankle device (**A**). Their goal was to exert an ankle dorsiflexion movement that reversed at the spatiotemporal target (9° movement in 180 ms). Older adults exhibited impaired endpoint control (**B**) and altered muscle activity for the Tibialis Anterior (TA) and Soleus (SOL) muscles (**C**). Specifically, older adults exhibited similar TA muscle activity but relatively more SOL muscle activity compared with young adults.

Participants performed unloaded, fast, reverse-at-target goal-directed movements that involved accurately matching the peak displacement of the limb to a target. To achieve this movement with accuracy participants had to reverse ankle dorsiflexion with plantarflexion at the spatiotemporal target (9° spatial target and 180 ms time to reach the target; Fig. 1). The task contained the following three phases: (1) GET READY, (2) MOVE, and (3) FEEDBACK. The GET READY phase began by the presence of a red target on the monitor for 2 s. This was a cue for the subjects to be ready for the MOVE phase. The MOVE phase began when the red target switched to a green target. This target color change was the cue for subjects to perform the goal-directed movement. The green target stayed on the monitor for 3 s, and subjects were instructed to initiate a movement at their convenience (not a reaction time task). The recording of the task began when the subject initiated the movement within the 3 s of the MOVE phase. We emphasized that to do this movement correctly, they would need to move their foot up towards the target (i.e. dorsiflexion) and then immediately move their foot back down (i.e. plantarflexion). If participants did not execute the downward portion of the parabola, (i.e. held their position at the target), their endpoint was recorded inaccurately and we marked the trial as a failed trial. After a failed trial, we repeated the instructions of the task to ensure proper performance. The FEEDBACK phase began at the end of each MOVE phase and lasted for 5 s. We provided the subjects with visual feedback of their movement trajectory trace relative to the targeted angle-time endpoint. The visual gain was kept constant at 1° (visual angle) for all trials^6,21^.

#### Data analysis

Data was analyzed off-line using custom-written programs in MATLAB (MathWorks, Natick, MA). We calculated the endpoint accuracy, endpoint variability, and activation of the tibialis anterior (TA) and soleus (SOL) muscle during the goal-directed task. We eliminated a very small number of trials (less than 5% of the total trials for both groups) based on the criteria that the force and time errors were within the 95th percentile of the data.

##### Endpoint control

To calculate the endpoint error we quantified the spatial and temporal errors. We quantified spatial error as the absolute deviation from the targeted peak displacement and quantified temporal error as the absolute deviation from the targeted time to peak displacement. The spatial error was normalized to the targeted peak displacement (Eq. 1) and the temporal error was normalized to the targeted time to peak displacements (Eq. 2). In addition, we quantified the trial-to-trial variability of the responses by quantifying spatial variability as the coefficient of variation [CV; (SD of response/mean response) × 100)] of the peak displacement and temporal variability as the CV of time-to-peak displacement. Therefore, the endpoint was characterized by the following: (1) spatial error; (2) temporal error; (3) spatial variability; and (4) temporal variability.

1 $$Spatial \,error \left( \% \right) = \frac{{{\text{peak }}\,{\text{displacement }}\,(^{{\text{o}}} )}}{{{\text{targeted }}\,{\text{peak }}\,{\text{displacement }}\,(^{{\text{o}}} )}}{ } \times 100,$$

2 $$Temporal\, error \left( \% \right) = \frac{{{\text{time }}\,{\text{to }}\,{\text{peak }}\,{\text{displacement }}\left( {{\text{ms}}} \right)}}{{{\text{targeted }}\,{\text{time }}\,{\text{to }}\,{\text{peak }}\,{\text{displacement }}\left( {{\text{ms}}} \right)}}{ } \times 100.$$

##### Muscle activity

The interference EMG was rectified and smoothed with a fourth-order Butterworth digital filter with a cutoff frequency of 6 Hz^22^. This filter was used to identify the amplitudes, onsets, and offsets of the EMG bursts for the primary agonist and antagonist muscles of the ankle. We quantified the burst activity as amplitude, time-to-peak, normalized (CV) trial-to-trial amplitude variability, and normalized (CV) trial-to-trial time-to-peak variability. The burst amplitude was the peak muscle activity between the burst onset and offset. The burst time-to-peak was the latency between burst onset and burst amplitude. The burst amplitude and time-to-peak variability was the coefficient of variation across trials.

#### Statistical analysis

We compared the endpoint control and muscle activity of young and older adults using a non-parametric independent samples Mann–Whitney U Test (mwU). The dependent variables were (1) spatial error, (2) temporal error, (3) spatial variability, (4) temporal variability, (5) EMG burst amplitude, (6) EMG burst time-to-peak amplitude, (7) EMG burst amplitude variability, and (8) EMG burst time-to-peak variability. We used bivariate correlation to determine the association between muscle activity and endpoint control. The goodness of fit of each regression was given by the squared correlation (R^2^)^23^. All statistical analyses were performed with the IBM statistics 21.0 statistical package (IBM, New York, NY). The α level for all statistical tests was 0.05. Data are reported as means ± SD in the text and as means ± SE in figures.

### Experiment 2

#### Participants

Fifteen young adults (mean 20.5 ± 1.4 years, range 18–23 years, 9 women) volunteered to participate in this study. All participants reported being healthy without any known neurological or orthopedic disorders with an average Montreal Cognitive Assessment score of 28.1 ± 1.2^24^. Participants performed that task using the non-dominant foot as assessed with the Waterloo Footedness Questionnaire^13^. The Institutional Review Board at the University of Florida approved the procedures, and participants signed a written informed consent before participating in the study.

#### Experimental approach

The experimental approach for Experiment 2 was identical to that of Experiment 1 with the exception of the task. Although the task used in both experiments involved ballistic targeted contractions, we opted to use an isometric task for Experiment 2. Our goal was to study the effect of the altered motor plan on endpoint control. To minimize the influence of the stretch-reflex (which could have contributed to the findings of Experiment 1), we asked participants to perform the targeted contractions with isometric ballistic ankle targeted contractions.

**Figure 2:**
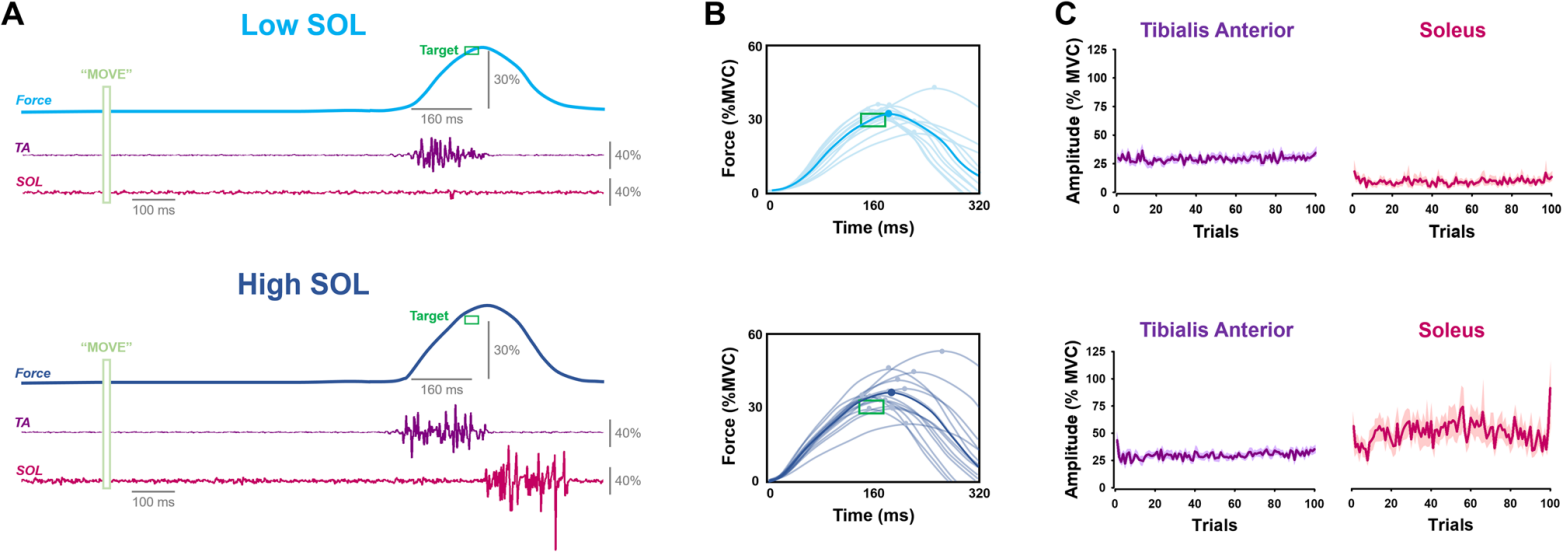
Isometric endpoint control and muscle activation in young adults with altered strategies. Participants performed a goal-directed ankle dorsiflexion contraction with the non-dominant foot that was restrained in an ankle device (**A**). Their goal was to exert an ankle dorsiflexion contraction that reversed at the spatiotemporal target (30% force in 160 ms). We asked participants to perform these goal-directed contractions using strategies similar to what was observed in Experiment 1 between young and old (see Fig. 1). Using a Low SOL coordination pattern (i.e. similar to young), participants performed the task by activating their TA and immediately relaxing. Using a High SOL coordination pattern (i.e. similar to old), participants performed the task by activating the TA during the upward portion followed by immediate activation of the SOL during the downward portion. Using the High SOL coordination pattern, participants exhibited impaired endpoint control (**B**) and altered muscle activity for the Tibialis Anterior (TA) and Soleus (SOL) muscles (**C**). Specifically, TA muscle activity was similar but SOL muscle activity was larger while using a High SOL coordination pattern compared to the Low SOL coordination pattern.

Each participant performed 100 trials of an isometric ankle dorsiflexion–plantarflexion goal-directed task using two separate coordination patterns. For the first pattern, termed **Low SOL**, we asked participants to exert a force parabola and match the peak of the force parabola with the force/time target (30% MVC and 160 ms) by performing the dorsiflexion portion of the task by activating their TA and reversing the contraction (plantarflexion) by relaxing the TA muscle. This would minimize the activation of the soleus muscle for the plantarflexion of the task and mimic the motor plan used by the young adults in Experiment 1. For the second pattern, termed **High SOL**, we asked participants to exert a force parabola and match the peak of the force parabola with the force/time target (30% MVC and 160 ms) by performing the dorsiflexion portion of the task by activating their TA and reversing the contraction (plantarflexion) by activating their soleus muscle (Fig. 2). This coordination pattern would mimic the motor plan used by the older adults in Experiment 1. We counterbalanced the order of the two experimental conditions with 8 participants performing the High SOL pattern first followed by the Low SOL pattern. The other 7 participants performed the Low SOL pattern first followed by the High SOL pattern.

#### Experimental setup

Each participant seated comfortably in an upright position and faced a 32 inch monitor (Sync MasterTM 320MP-2, Samsung Electronics America, Ridgefield Park, NJ, USA) that was located 1.25 m away at eye level. The hip joint was flexed to 90°, abducted by 10°, and knee was flexed 90°. The foot rested on a customized foot device with an adjustable footplate that we secured by straps over the metatarsals to ensure an isolated dorsiflexion of the ankle. The monitor displayed the contraction produced by ankle dorsiflexion using a custom-written program in Matlab (Math WorksTM Inc., Natick, MA, USA) and all participants affirmed that they could see the display clearly.

#### Measurements

##### Ankle force

The maximum voluntary force exerted and the force exerted during the goal-directed contraction were measured using a force transducer (Model MB-100, Interface, Scottsdale, AZ, USA) that was located in parallel with the force direction on the customized foot device. The ankle force signal was sampled at 1000 Hz with a NI-DAQ card (Model USB6210, National Instruments, Austin, TX, USA), and stored on a personal computer. The raw force signals were smoothed using a 4th order Butterworth digital filter with an optimal cut-off frequency of 20 Hz. The beginning of each trial was defined by the time at which the force signal equaled 5% of the peak force for that trial.

##### EMG

EMG measurements in Experiment 2 were identical to that of Experiment 1.

#### Experimental tasks

##### MVC task

The MVC task in Experiment 2 was identical to that of Experiment 1.

##### Goal-directed task

Participants performed goal-directed contractions that involved accurately matching the peak force of the limb to a target. To achieve this contraction with accuracy participants had to reverse ankle dorsiflexion with plantarflexion at the force/time target (30% MVC force target and 160 ms time to reach the target; Fig. 2). The GET READY phase began by the presence of a red target on the monitor for 100 ms, the green target stayed on the monitor for 1 s, and the FEEDBACK phase lasted for 1 s. All other goal-directed parameters were identical to the ones described for Experiment 1.

#### Data analysis

Data were analyzed off-line using custom-written programs in Matlab. We analyzed force and time endpoint accuracy and variability during the ankle dorsiflexion goal-directed task. In addition, we examined activation of the TA and SOL muscles (burst). We eliminated a very small number of trials (less than 5% of the total trials for both groups) based on the criteria that the force and time errors were within the 95th percentile of the data.

##### MVC

We quantified the maximal force produced during the MVC task. From the MVC trials, we selected the trial with the highest force as a representation of the participant’s maximal force capacity. We used this maximal value to normalize the force target for each participant.

##### Endpoint control

Similarly to Experiment 1, we quantified force error as the absolute deviation from the targeted peak force and quantified temporal error as the absolute deviation from the targeted time to peak force. In addition, we quantified the trial-to-trial endpoint variability of the performance by quantifying the CV of peak force and time-to-peak force. Therefore, endpoint accuracy and variability were characterized by the following: (1) force error; (2) temporal error; (3) force variability; and (4) temporal variability.

##### Muscle activity

We quantified the activation of the TA and SOL muscles as described in Experiment 1.

#### Statistical analysis

We compared the endpoint control and muscle activity of the two coordination patterns using paired t-tests. The dependent variables were: (1) force error, (2) force variability, (3) temporal error, (4) temporal variability, (5) TA amplitude, (6) TA time-to-peak, (7) TA amplitude variability, and (8) TA time-to-peak variability. The alpha level for all statistical tests was 0.05, which was corrected for multiple comparisons. We used bivariate correlations to determine the association between muscle activity and endpoint control. The goodness of fit of each regression was given by the squared correlation (R^2^)^23^. We performed all statistical analyses with the IBM statistics 24.0 statistical package (IBM, Armonk, NY). Data are reported as means ± SD within the text and as means ± SE in the figures.

## Results

### Experiment 1

#### Endpoint control

**Figure 3:**
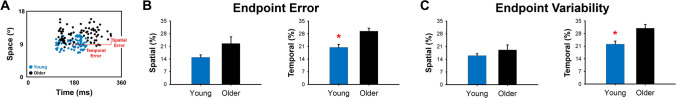
Age-associated differences in movement endpoint control. Representative example for a young and an older adult (**A**). On average, older adults exhibited similar spatial error but greater temporal error relative to young adults (**B**). Furthermore, older adults exhibited greater temporal variability but similar spatial variability (**C**). *p < 0.05.

We characterized endpoint control in young and older adults by quantifying spatial and temporal error and variability (Fig. 3A). The spatial error (mwU = 47, p = 0.065; Fig. 3B) and spatial variability (mwU = 37, p = 0.32; Fig. 3C) were not significantly different for the two groups. However, older adults exhibited significantly greater temporal error (mwU = 60, p = 0.001; Fig. 3B) and temporal variability (mwU = 56, p = 0.005; Fig. 3C). These findings suggest that older adults exhibit impaired endpoint control, as demonstrated from greater temporal errors and variability.

#### TA and SOL muscle activity

**Figure 4:**
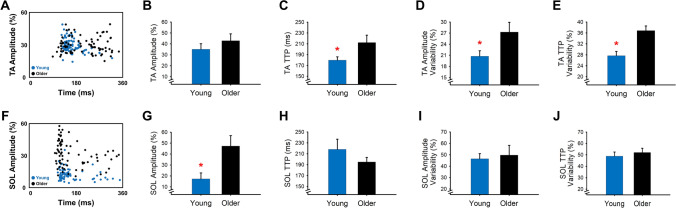
Age-associated differences in muscle activation. Representative example of TA muscle activity (**A**) and SOL muscle activity (**F**) for a young and an older adult. Relative to young adults, older adults had similar normalized TA amplitude (**B**) but greater TA time-to-peak (TTP; **C**), TA amplitude variability (**D**), and TA time-to-peak variability (**E**). Furthermore, older adults exhibited greater normalized SOL amplitude (**G**) but similar SOL time-to-peak (**H**), SOL amplitude variability (**I**), and SOL time-to-peak variability (**J**). *p < 0.05.

We characterized the TA (Fig. 4A) and SOL (Fig. 4F) muscle activity for young and older adults by quantifying the EMG burst amplitude and time-to-peak, as well as their trial-to-trial variability. We found that the TA EMG amplitude was not significantly different between young and older adults (mwU = 39, p = 0.25; Fig. 4B), but the SOL EMG amplitude was significantly greater in older adults compared with young adults (mwU = 58, p = 0.003; Fig. 4G). In addition, older adults exhibited significantly greater TA time-to-peak EMG (mwU = 54, p = 0.011; Fig. 4C), but not significantly different SOL time-to-peak EMG (mwU = 22, p = 0.16; Fig. 4H). Furthermore, older adults exhibited greater TA EMG amplitude variability (mwU = 49, p = 0.041; Fig. 4D) and TA time-to-peak EMG variability (mwU = 64, p < 0.001; Fig. 4E) but similar SOL EMG amplitude variability (mwU = 30, p = 0.44; Fig. 4I) and SOL time-to-peak EMG variability (mwU = 35, p = 0.40; Fig. 4J). These findings suggest that older adults exhibit an altered coordination pattern relative to young adults.

**Figure 5:**
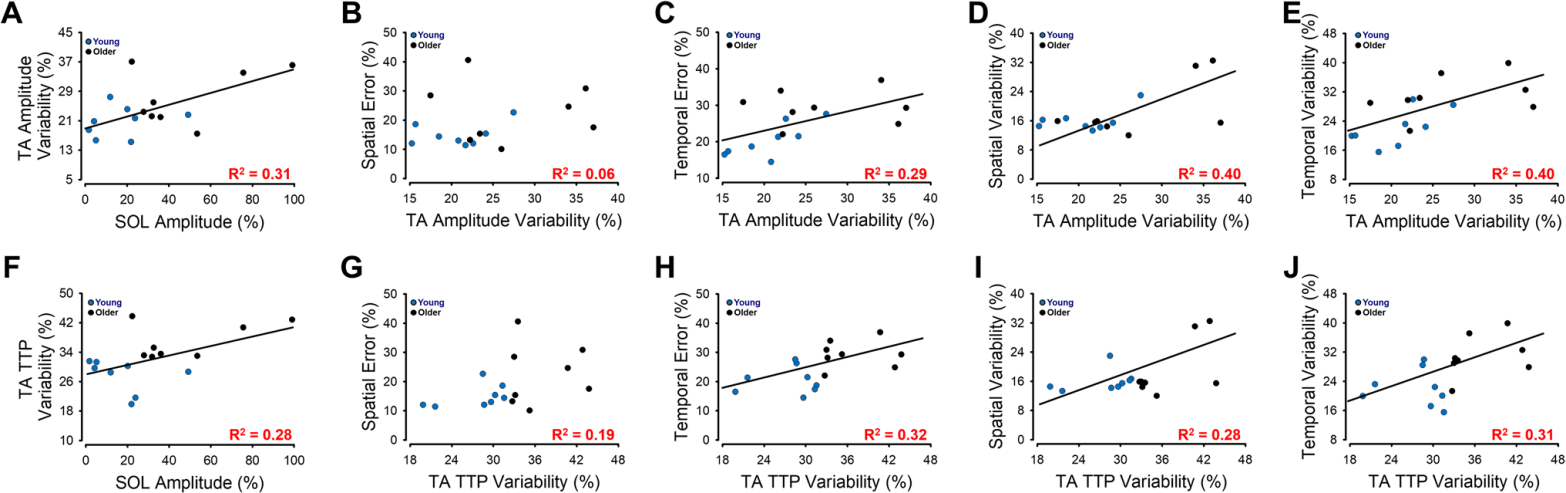
TA muscle activity associations with SOL muscle activity and endpoint control for young and older adults. SOL muscle activity significantly and positively associated with TA amplitude variability (**A**). Consequently, TA amplitude variability did not significantly associate with spatial error (**B**) but significantly and positively associated with temporal error (**C**), spatial variability (**D**), and temporal variability (**E**). Similarly, SOL muscle activity significantly and positively associated with TA time-to-peak (TTP) variability (**F**). Moreover, TA TTP variability did not significantly associate with spatial error (**G**) but significantly and positively associated with temporal error (**H**), spatial variability (**I**), and temporal variability (**J**).

To determine how the amplitude of SOL muscle activity influenced the TA muscle activity, we examined the correlation between the SOL EMG amplitude with the TA EMG amplitude and TA time to peak EMG, as well as with their variability (CV). The associations between SOL EMG amplitude with the TA EMG amplitude and SOL EMG amplitude with the TA time to peak EMG were not significant (p > 0.1). In contrast, the SOL EMG amplitude significantly and positively associated with the variability of the TA EMG amplitude (R^2^ = 0.31, p = 0.026; Fig. 5A) and the variability of TA time to peak EMG (R^2^ = 0.28, p = 0.036; Fig. 5F). These findings suggest that the greater SOL activity in older adults increases their TA muscle activity variability.

#### TA EMG variability and endpoint control

To determine how the age-associated increase in TA muscle activity variability influences endpoint control we examined the correlation between the TA EMG variability and measures of endpoint control. The TA EMG amplitude variability was not associated with the spatial error (p > 0.1; Fig. 5B) but positively associated with temporal error (R^2^ = 0.29, p = 0.03; Fig. 5C), spatial variability (R^2^ = 0.4, p = 0.009; Fig. 5D), and temporal variability (R^2^ = 0.4, p = 0.009; Fig. 5E). Similarly, the TA time to peak EMG variability was not associated with the spatial error (p = 0.09; Fig. 5G) but positively associated with temporal error (R^2^ = 0.29, p = 0.03; Fig. 5H), spatial variability (R^2^ = 0.4, p = 0.009; Fig. 5I), and temporal variability (R^2^ = 0.4, p = 0.009; Fig. 5J). These findings suggest that the increased TA EMG variability in older adults impairs endpoint control.

### Experiment 2

#### Endpoint control

**Figure 6:**
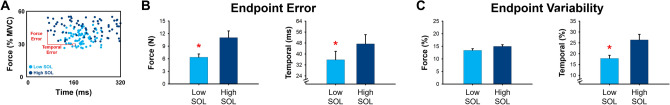
Endpoint control for young adults using a Low SOL and High SOL coordination pattern. A Low SOL coordination pattern resembled the one used by young adults, whereas a High SOL coordination pattern resembled the one used by older adults in Experiment 1. Representative example for young adults using a Low SOL and High SOL coordination pattern (**A**). When young adults performed the goal-directed ankle dorsiflexion contraction using a High SOL coordination pattern, they exhibited greater force and temporal (**B**) error. Furthermore, young adults exhibited greater temporal variability but similar force variability with the High SOL coordination pattern (**C**). *p < 0.05.

We characterized the endpoint control for two muscle coordination patterns (Low SOL vs. High SOL; Fig. 6A) in young adults by quantifying force and temporal error and variability. Young adults exhibited greater force error (t = − 3.94, p = 0.001; Fig. 6B) but not significantly different force variability (t = − 1.72, p = 0.11; Fig. 6C) for the High SOL relative to the Low SOL coordination pattern. In addition, the young adults exhibited greater temporal error (t = − 3.18, p = 0.006; Fig. 6B) and temporal variability (t = − 3.46, p = 0.004; Fig. 6C) for the High SOL coordination pattern. These findings suggest that when young adults use a muscle coordination pattern that mimics the one used by older adults in Experiment 1 (High SOL), they exhibit impaired endpoint control.

#### TA and SOL muscle activity

**Figure 7:**
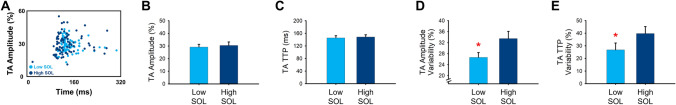
Muscle activity for the tibialis anterior and soleus muscles for young adults using a Low SOL and High SOL coordination pattern. Representative example of TA muscle activity (**A**) for young adults using a Low SOL and High SOL coordination pattern. Using a High SOL coordination pattern, young adults exhibited similar normalized TA amplitude (**B**) and TA time-to-peak (TTP; **C**), but greater TA amplitude variability (**D**), and TA time-to-peak variability (**E**) relative to a Low SOL coordination pattern. *p < 0.05.

Similar to Experiment 1, we characterized the TA (Fig. 7A) and SOL muscle activity for the two coordination patterns by quantifying the EMG burst amplitude and time-to-peak, as well as their normalized trial-to-trial variability. Young participants were successful in performing the two coordination patterns as instructed. They activated the SOL more during the High SOL (50.8 ± 38.1%) than the Low SOL (9.5 ± 9.7%) coordination pattern (t = − 4.65, p < 0.01). The TA amplitude (t = − 0.94, p = 0.37; Fig. 7B) and time-to-peak (t = − 0.51, p = 0.62; Fig. 7C) was not significantly different for the two coordination patterns. In contrast, the TA EMG amplitude variability (t = − 3.27, p = 0.006; Fig. 7D) and TA time-to-peak EMG variability (t = − 2.46, p = 0.027; Fig. 7E) was significantly greater for the High SOL coordination pattern. These findings suggest that the variability of the TA muscle activity is greater when young adults use a High SOL than a Low SOL coordination pattern.

**Figure 8:**
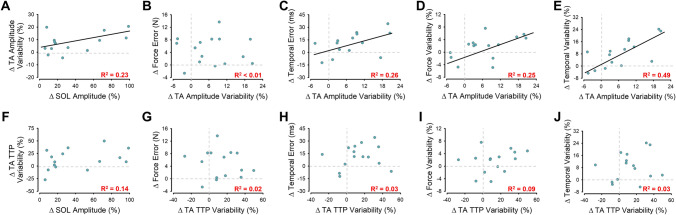
TA muscle activity associations with SOL muscle activity and endpoint control for young adults using a Low SOL and High SOL coordination pattern. Upon removal of an outlier, the association between the change in SOL muscle activity with the change in TA amplitude variability becomes significant (**A**). Consequently, the change in TA amplitude variability did not significantly associate with the change in force error (**B**) but significantly and positively associated with temporal error (**C**), force variability (**D**) and temporal variability (**E**). In contrast, the change in SOL muscle activity did not associate with the change in TA time-to-peak (TTP) variability (**F**) and the change in TA TTP variability did not associate with any measures of endpoint control (**G**–**J**).

To determine how the increase in the amplitude of SOL muscle activity with the dorsiflexion–plantarflexion pattern changed the TA muscle activity we examined the correlation between the change in SOL EMG amplitude with the changes in TA EMG amplitude and TA time to peak EMG, as well as with their variability. Although all correlations were not statistically significant (p > 0.1), it was clear that the change in variability measures of TA activity positively related to the change in SOL EMG amplitude. Removing a single outlier value, the association between the change in SOL EMG amplitude and TA EMG amplitude variability (R^2^ = 0.23, p = 0.04; Fig. 8A) became significant. These findings suggest that by increasing the SOL activity with the dorsiflexion–plantarflexion increases the TA muscle activity variability.

#### TA EMG variability and endpoint control

To determine how the increased TA muscle activity variability influences endpoint control, we examined the correlation between the increase in TA EMG variability and the change in measures of endpoint control. The increased TA EMG amplitude variability was not associated with the force error (p > 0.1; Fig. 8B) or the temporal error (p > 0.1; Fig. 8C), but positively associated with force variability (R^2^ = 0.25, p = 0.03; Fig. 8D), and temporal variability (R^2^ = 0.49, p = 0.009; Fig. 8E). The TA time to peak EMG variability was not associated with any of the endpoint measures (p > 0.1; Fig. 8F–J). These findings suggest that the increased TA EMG variability with the coordination pattern that mimics the one used by older adults impairs endpoint control.

## Discussion

Here, we aimed to understand if older adults select a unique motor plan that is detrimental to endpoint control. We performed two experiments that used ankle dorsiflexion–plantarflexion ballistic contractions that reversed at the target. In the first experiment, we found that the coordination pattern (motor plan) differed for young and older adults. Older adults used significantly greater soleus muscle activity to reverse the ankle movement (high SOL pattern) than young adults (low SOL pattern). Higher soleus activity was associated with greater TA muscle activity variability that consequently impaired endpoint control. In Experiment 2, we asked a distinct group of young adults to exert accurate ballistic ankle contractions either by using a pattern that mimicked older adults (High SOL) or young adults (Low SOL) from Experiment 1. When young adults used the pattern observed in older adults (High SOL), they exhibited greater TA muscle activity variability and impaired endpoint control relative to the pattern observed in young adults (Low SOL). These findings provide strong evidence that older adults select a motor plan that is detrimental to endpoint control.

### Motor planning

Motor planning is defined as the cognitive processing of organizing appropriate motor commands to execute specific movement goals^25,26^. It is integral for the accurate execution and learning of voluntary movements^27^. We constantly make decisions about how to perform accurate movements that take into account the state of the motor system and the environment^28^. Motor planning is also critical for motor learning, as memory formation of the particular motor task needs to adapt the motor plan^4,27^. For example, Sheahan et al. asked participants to perform a follow-through movement from a starting location to a central target with a velocity-dependent force field applied during the movement. They found that a ‘planning only’ group, in which participants viewed a secondary target to move towards but disappeared mid-movement, showed adaptation across multiple movements. In contrast, an ‘execution only’ group, in which participants only viewed the central target but a secondary target appeared mid-movement, showed no adaptation across multiple movements. Thus, they concluded that no learning occurs if different movements are executed but not planned prior to movement initiation^27^.

Here, we used muscle coordination during targeted ballistic ankle contractions as an index of the motor plan. We have used this experimental model in the past to study the motor plan of healthy older adults^1,2,4,5^ and patients with movement disorders^11,19^. Targeted ballistic contractions (< 200 ms) are primarily controlled by preplanned descending cortical commands that are not influenced by online feedback^5,7–10^. Adjustments to the motor plan occur only across trials. The execution of the motor plan is important beyond discrete ballistic contractions. Online visuomotor (and proprioceptive) corrections during tracing tasks are the result of continuously executed motor plans (updating of the motor command in real time to improve performance). For example, providing visual guidelines during a constant tracing task allowed participants to reduce variability relative to a visually unrestricted condition^29^. Thus, the motor plan and its execution seems to be critical for the performance and learning of various motor tasks^30^. In this study, our purpose was to determine if the selected motor plan differs for young and older adults and if it has any consequences to endpoint control.

### Age-associated differences in the motor plan

There is strong evidence that the neural activation of muscles differs for young and older adults^1,2,4,5,31,32^. For example, older adults exhibit greater motor unit discharge rate variability during tracing contractions^31,32^ and stronger modulation of muscle activity from 13 to 60 Hz during targeted contractions. Most critical to this study, there is evidence that muscle coordination differs for young and older adults for pre-planned ballistic targeted contractions. The current hypothesis is that older adults use alternative temporal parameters (i.e. timing of EMG activation) when planning ballistic contractions^1,2,4,5^. For example, when older adults aimed to produce accurate ballistic contractions that reverse at the target, they exhibited a longer delay between the dorsiflexor and plantarflexor muscles for an ankle movement^1^ and longer delay between the biceps brachii and triceps brachii for an elbow movement^2^. Although these findings suggest that the temporal parameters change between the coordinated muscles, little is known about the muscle amplitude parameters of the motor plan.

Our findings support the hypothesis that older adults select a unique motor plan that is detrimental to endpoint control. Specifically, in experiment 1, we found that, on average, older adults activate the soleus muscle (dorsiflexion) about three times as much as young adults (48% vs. 17%). This finding suggests that age-associated differences occur beyond the temporal characteristics of the coordinated muscles. Rather, the motor plan (coordination) to accomplish this targeted dorsiflexion–plantarflexion ballistic contraction differs for young and older adults. The underlying reason for older adults selecting this detrimental motor plan remains unclear. With aging, the connectivity^33^ and morphology^34^ of cortical centers change. For example, older adults exhibit reduced white matter ^35^ and grey matter^36^ volume, and weaker frontal-striatal connectivity compared with young adults^33^. In addition, the number and quality of cortical projections to the muscle decreases with age^37,38^. Therefore, the selection of a unique motor plan to accomplish targeted movements in older adults may be a compensation strategy due to the changes in the central and peripheral motor system. Regardless of the exact reason, Experiment 1 provides evidence that older adults select an altered motor plan than young adults, which relates to endpoint control impairments.

How does the over activation of the soleus muscle in older adults, which temporally occurs after the target, impair endpoint control? According to our findings, a motor plan with greater activity of the SOL muscle increases the TA variability, the muscle that moves the foot towards the target. This finding suggests that when the motor plan is structured to include a significant SOL activity, it changes the activity of the primary mover (TA).

To confirm that this unique motor plan is detrimental to endpoint control, we performed a distinct experiment that manipulated the coordination pattern. In Experiment 2, we recruited young adults so we could quantify only the effects of the coordination pattern and not the effects of age-associated physiological changes. We find that when young adults performed a ballistic targeted ankle contraction with a coordination pattern that mimicked the one used by older adults (High SOL) in Experiment 1, they exhibited impaired endpoint control. Young adults exhibited similar changes to what was observed in Experiment 1 for older adults relative to young adults. Specifically, with the High SOL coordination pattern, young adults significantly increased the TA muscle activity variability and exhibited approximately double the spatial and temporal error than when performing the same task with the Low SOL pattern. Thus, the findings from Experiment 2 confirmed that a pre-planned coordination pattern that increases the activation of soleus is detrimental to endpoint control.

### Limitations

Although we provide strong support that older adults use a unique motor plan to accomplish targeted contractions, our findings are limited to 100 practice trials. It is possible that with extensive practice, older adults will inherently modify their motor plan to be similar to that of young adults. Future work is needed to determine if the age-associated differences in motor plan remain even after extensive practice. Additionally, it is unclear if sex differences could contribute to group differences. There is evidence that young women exhibit differences in the temporal parameters of coordinated muscles that likely contribute to greater inaccuracy relative to young men^39^. However, the relative amplitudes of the coordinated muscles was not different^39^. In contrast, the literature on sex differences in older adults for endpoint control is scarce. Finally, another important limitation of our study is that we don’t provide any direct measures of brain activity associated with motor planning. More studies are needed to explore how brain areas are activated differently for young and older adults during these ballistic contractions.

Our findings support the hypothesis that older adults select a unique motor plan that is detrimental to endpoint control. Our evidence comes from two distinct experiments. The first experiment demonstrated a unique motor plan for older adults to perform targeted movements that was associated with impaired endpoint control. The second experiment, confirmed that the selected motor plan by older adults is detrimental to endpoint control. Even when young adults used a coordination pattern that was selected by older adults, they exhibited similar impairments in endpoint control. Although the exact reason of why older adults select this altered motor plan remains unknown, we provide strong support to the notion that age-associated changes in motor planning impair movement control. The findings could have implications in the design of rehabilitation protocols to enhance the control of various types of movements and motor learning in older adults.
